# Quantitative evaluation of a cone‐beam computed tomography–planning computed tomography deformable image registration method for adaptive radiation therapy

**DOI:** 10.1120/jacmp.v8i4.2432

**Published:** 2007-11-05

**Authors:** Joshua D. Lawson, Eduard Schreibmann, Ashesh B. Jani, Tim Fox

**Affiliations:** ^1^ Department of Radiation Oncology Emory University Atlanta Georgia U.S.A.

**Keywords:** Image‐guided radiation therapy, deformable image registration, adaptive radiotherapy

## Abstract

Deformable (non‐rigid) registration is an essential tool in both adaptive radiation therapy and image‐guided radiation therapy to account for soft‐tissue changes during the course of treatment. The evaluation method most commonly used to assess the accuracy of deformable image registration is qualitative human evaluation. Here, we propose a method for systematically measuring the accuracy of an algorithm in recovering artificially introduced deformations in cases of rigid geometry, and we use that method to quantify the ability of a modified basis spline (B‐Spline) registration algorithm to recover artificially introduced deformations. The evaluation method is entirely computer‐driven and eliminates biased interpretation associated with human evaluation; it can be applied to any chosen method of image registration.

Our method involves using planning computed tomography (PCT) images acquired with a conventional CT simulator and cone‐beam computed tomography (CBCT) images acquired daily by a linear accelerator–mounted kilovoltage image system in the treatment delivery room. The deformation that occurs between the PCT and daily CBCT images is obtained using a modified version of the B‐Spline deformable model designed to overcome the low soft‐tissue contrast and the artifacts and distortions observed in CBCT images. Clinical CBCT images and contours of phantom and central nervous system cases were deformed (warped) with known random deformations. In registering the deformed with the non‐deformed image sets, we tracked the algorithm's ability to recover the original, non‐deformed set. Registration error was measured as the mean and maximum difference between the original and the registered surface contours from outlined structures. Using this approach, two sets of tests can be devised. To measure the residual error related to the optimizer's convergence performance, the warped CBCT image is registered to the unwarped version of itself, eliminating unknown factors such as noise and positioning errors. To study additional errors introduced by artifacts and noise in the CBCT image, the warped CBCT image is registered to the original PCT image.

Using a B‐Spline deformable image registration algorithm, mean residual error introduced by the algorithm's performance on noise‐free images was less than 1 mm, with a maximum of 2 mm. The chosen deformable image registration model was capable of accommodating significant variability in structures over time, because the artificially introduced deformation magnitude did not significantly influence the residual error. On the second type of test, noise and artifacts reduced registration accuracy to a mean of 1.33 mm and a maximum of 4.86 mm.

The accuracy of deformable image registration can be easily and consistently measured by evaluating the algorithm's ability to recover artificially introduced deformations in rigid cases in which the true solution is known *a priori.* The method is completely automated, applicable to any chosen registration algorithm, and does not require user interaction of any kind.

PACS numbers: 87.57.Gg, 87.57.Ce, 87.62.+n

## I. INTRODUCTION

During the course of radiation therapy, daily changes such as bowel gas and bladder filling alter the soft‐tissue landscape within a patient's anatomy,^(^ ^1^ ^–^ ^3^ ^)^ adding to the more gradual changes in tumor or target‐structure volume and shape over the course of treatment. Positional changes are usually assessed using electronic portal images acquired either weekly or, less commonly, immediately before delivery of each fraction. A common practice is to register these verification images with digitally reconstructed radiographs generated from the planning computed tomography (PCT) data. If a significant difference is observed, corrections are made by shifting the isocenter until the bony anatomy or some other fiducial marker of the two images is grossly aligned. A typical case is prostate treatment, in which translations as large as 5 – 9 mm are reportedly needed to compensate for changes in bladder filling or rectal gas and stool.^(4)^

However, organs are not rigid, and a simple translation cannot capture the full extent of the anatomic changes. The true deformation is three‐dimensional (3D) by nature, because organs change in both shape and location. Shape changes cannot be fully assessed from projected images and cannot be fully corrected by simple translation. Because the true target deformation at the time of treatment is unknown, margins large enough to accommodate expected target shift and deformation are used to ensure coverage in all treatment sessions. These margins lead to reductions in the maximum deliverable target dose by as much as 13%^(5)^ because of the increasing difficulty in escalating the target dose without exceeding the dosimetric limits of neighboring critical organs.

Margins could potentially be reduced if an accurate description of the patient geometry at the time of each treatment fraction were available,^(5)^ because the dose received by each voxel could be tracked^(^ ^6^ ^–^ ^12^ ^)^ and included in the dose optimization.^(^ ^13^ ^–^ ^15^ ^)^ Adaptive radiotherapy (ART) is an evolving area of much interest, aimed at developing techniques by which a course of radiation therapy could continually be monitored and modified to reflect the anatomic changes known to occur.

More recently, cone‐beam CT (CBCT) using a kilovoltage imaging system mounted on the linear accelerator has emerged as a promising technique for accomplishing soft‐tissue registration. A CBCT image of the patient on the treatment table can be acquired in about 60 seconds, just before delivery of each treatment fraction. Essential to incorporating daily changes in the treatment process is the use of deformable registration. Deformable registration involves voxel‐dependent modeling in addition to the three displacement and three rotation parameters of rigid registration. Considerably more complex than rigid registration, deformable registration offers the potential for warping a structure's surfaces to reflect changes over time and could be used to automatically warp structures contoured at the time of simulation to their location just before delivery of each fraction.

Accomplishing this registration with accuracy is essential, because precise radiotherapy cannot be delivered without confidence in the deformation model's ability to incorporate small organ changes. Technically, deformable registration is an optimization problem that aims to recover unknown parameters of a transformation and to correlate anatomic features observed in two different images of the same subject. An iterative process is usually employed to modify the transformation parameters until an optimal match is found between input images.

Several techniques have been used to accomplish deformable image registration, including the fluid‐flow approach, the finite element approach, and splines. The spline technique relies on manual selection of control points (“nodes”). These chosen points correspond to distinct features of the imaged organ or organs. The displacement of any other point is then determined by interpolation of the displacement for the nearest control points. This technique has a validated ability to accurately recover artificially introduced intramodality deformations.^(16)^ More recently, a simpler technique has been proposed. In contrast to the spline algorithm, the basis spline (B‐Spline) technique uses nodes that are selected automatically, removing reliance on operator skill. B‐Spline is the technique used in this report.

Quality assurance of image registration methods is difficult to perform for all automated systems. The ideal image registration method would provide an uncertainty value on the accuracy of the image registration. This uncertainty could then be incorporated into the radiation treatment planning process by the addition of margins to the registered functional‐image structures, indicating the spatial uncertainty of the image match.

Because the true values of the transformation parameters are unknown in clinical cases with deforming anatomy, surrogates are used to evaluate accuracy. Failure or success is commonly judged qualitatively by either visual inspection or a comparison of the automatically‐generated and human‐delineated contours. Both methods introduce bias, given that centimetric errors have been reported in human judgment of the optimal match.^(17)^ Visual inspection compares the registration in just a few image regions and thus will not verify the solution globally. Contour‐based evaluation mimics clinical objectives, but human segmentation of soft tissue is inaccurate, with 10% – 18% inter‐observer variability^(18)^ with the use of CT images, which increases with the use of megavoltage CT images,^(19)^ because soft‐tissue organs can be difficult to distinguish.

Consequently, measuring registration accuracy against human‐produced baselines provides a qualitative rather than quantitative evaluation because of the intrinsic inaccuracy in establishing the baseline. Knowing the true deformation field permits a better evaluation of the algorithm, because the registration results can be compared directly with the true deformation field rather than with manually produced surrogates. Evaluation of registration accuracy is independent of the registration method used and therefore applicable to rigid and to deformable registration methods alike, no matter the algorithm employed.

The purpose of the present work was to use a situation in which the true transformation parameters were known to propose an evaluation scheme for a deformable image registration method. The main idea was to introduce random artificial deformations of trackable intensity in clinical images with rigid geometry. Because this environment is controlled, the true solution that should be found by the registration is known, *a priori*, to correspond to a non‐deformation field. To evaluate a deformable registration algorithm, we initially performed CT‐to‐CT registration of an image and structures to an artificially deformed version of the same image and structures. This step quantified the error intrinsic to the algorithm. In the second portion of the present work, we similarly performed a non‐rigid registration of CBCT‐to‐CT images and evaluated the effect of noise on the registration algorithm. Finally, we undertook a case study to test the algorithm in a clinical setting.

## II. METHODS

### A. Image acquisition and contouring

We obtained CT and CBCT images of a Quasar Body phantom (Modus Medical Devices, London, ON).^(20)^ This Lucite phantom is designed for non‐dosimetric quality assurance.

The CT images were obtained using a GE LightSpeed scanner (GE Medical Systems, Milwaukee, WI), with a 2.5‐mm slice thickness. The CBCT images were obtained using the on‐board imaging (OBI) system (Varian Medical Systems, Palo Alto, CA) attached to the gantry of the linear accelerator. The OBI system consists of an X‐ray tube (model G242, 0.4‐ and 0.8‐mm focal spots, 14‐degree anode angle, 800 kJ/h: Varian Medical Systems, Salt Lake City, UT) and an amorphous‐silicon imaging panel (model PaxScan 4030CB: Varian Medical Systems, Salt Lake City) attached at 90 degrees to the gantry with Exact robotic arms (Varian Medical Systems, Baden, Switzerland). Three motorized pivot points on the robotic arms allow the OBI to be remotely extended or retracted from the control console station.

After the CBCT and CT scans of the phantom had been performed, the image sets were sent for structure delineation by the Eclipse software package (Varian Medical Systems, Palo Alto).

The 30×20×8−cm Quasar Body phantom has three cylindrical inserts, each 8 cm in length and 8 cm in diameter. These inserts contain various structures designed to provide a range of surface contours and edges. The first insert contains a 5‐mm cylinder and a 10‐mm cylinder (each 5 cm in length) and a 20‐degree air wedge. The middle insert contains a 60‐degree air wedge and spheres 40 mm, 20 mm, and 10 mm in diameter. The third insert contains a 125‐cm^3^ cube. The phantom additionally contained two contoured rods (2.5 cm in diameter and 1.8 cm in length), one air and one solid. Table 1 shows the Hounsfield units (HU) of each structure. Each of these structures was manually contoured on each slice of the CT and CBCT image sets by a trained physicist (ES). The body of the phantom was contoured using the automatic body search tool in Eclipse.

**Table 1:** Mean and maximum structure deformations introduced and remaining after registration for artificial deformations of ±20 mm

			Offset (mm)			
			After deformation		After registration	
Structure	Size	HU	Mean	Maximum	Mean	Maximum
Body		137	2.33	10.24	0.03	1.15
Cube	125 cm^3^	347	3.73	9.40	0.68	2.45
Sphere	40 mm	383	1.70	4.42	0.29	0.89
	20 mm	355	1.96	4.36	0.21	0.56
	10 mm	324	2.37	4.57	0.74	1.49
Air wedge	20 degrees	−1000	2.98	9.41	0.53	3.54
	60 degrees	−1000	3.20	9.29	0.21	1.46
Cylinder	10 mm	315	2.46	6.17	0.43	2.08
	5 mm	318	3.03	9.85	0.20	0.53
Solid rod	25 mm	735	2.50	7.10	0.15	1.37
Air rod	25 mm	−1000	2.06	6.49	0.25	2.01

### B. Software platform

The Insight Toolkit, an open‐source software toolkit sponsored by the National Library of Medicine and the National Institutes of Health, was used for deformable image registration in this investigation. The toolkit consists of an extendable library of template‐based code for many image registration, visualization, and segmentation algorithms. The Digital Imaging and Communications in Medicine (DICOM) protocol was used for image data communication between the CT scanner and the OBI system. The Insight Toolkit has a DICOM filter configured to read DICOM‐formatted image files. All calculations were performed on a standard personal computer with a Windows XP operating system (Microsoft, Redmond, WA), a Pentium 4 processor operating at 1.6 GHz, and 256 MB of random access memory.

### C. Image warping

**Figure 1:**
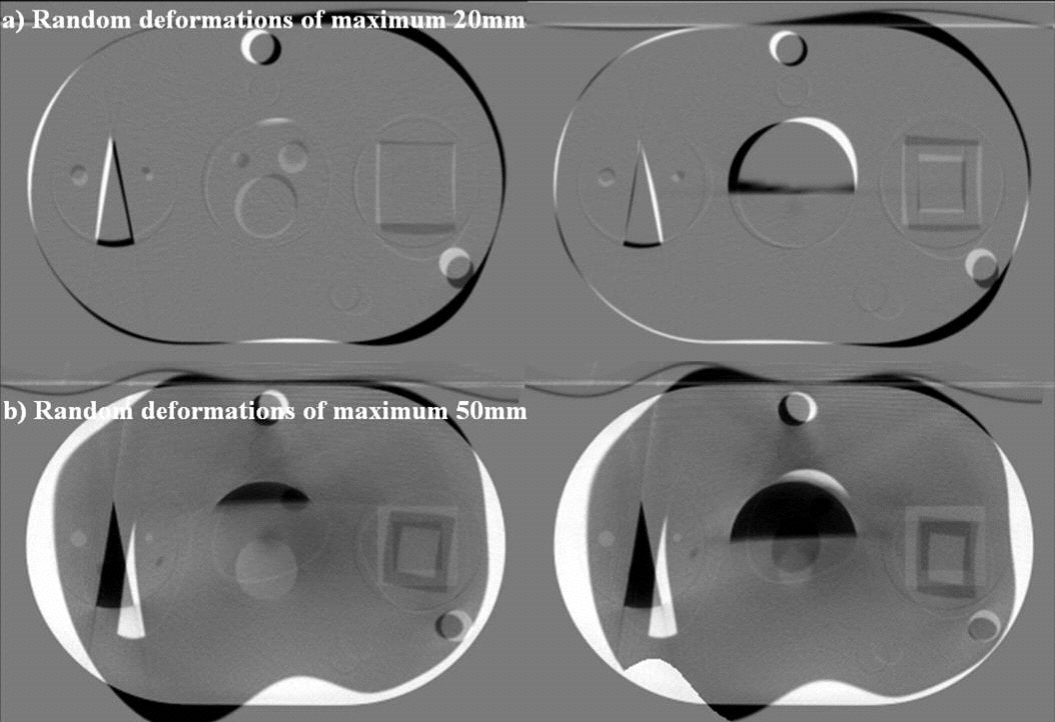
Subtraction images of the Quasar phantom (Modus Medical Devices, London, ON). Images with artificial deformations of (a) ±20 mm and (b) ±50 mm are overlaid on the original computed tomography image set. The scale has been set such that areas of pixel intensity agreement between the two images are gray, and areas with large positive or negative differences are shown in black or in white respectively.

Synthetic CT images with artificial deformations of both the native image and the segmented structures were created by assigning random transformation parameters at each B‐Spline node of the deformation model detailed in next subsection. Two levels of artificial deformations were introduced for maximum linear deformations of ±20 mm and ±50 mm in any direction (Fig. 1).

### D. Image co‐registration

The image that is being matched is typically called the fixed (“target”) image. The image whose coordinate system is being moved to match the fixed image is called the moving or floating image. Image registration is a three‐step process:

1. Use segmentation or classification to identify relevant contoured regions of interest in the volumes to be matched.
2. Define a similarity metric (“cost function”) to measure how well the two images are aligned.
3. Search for the best transformation to bring the two images into spatial alignment.

The PCT‐to‐CBCT registration is a quasi‐intermodality case. Although the underlying physical acquisition process is identical for the PCT and CBCT images, the acquisition geometry is different, causing the CBCT often to be corrupted by noise and artifacts. Moreover, the CBCT calibration procedure has its own intrinsic errors, with differences of up to 100 HU being observed for perirectal tissues and musculature in CBCT images.

From the deformable registration models available, we selected the noise‐tolerant B‐Spline technique^(21)^ combined with the Mattes formulation of the mutual information metric^(22)^ to permit multimodality registration as a more generous framework for the registration. The mathematical foundation of the B‐Spline model has been presented extensively in previous publications, and the performance and characteristics of the B‐Spline model have been intensively studied.^(^ ^23^ ^–^ ^25^ ^)^ The model has been applied to prostate CT–to–magnetic resonance or magnetic resonance spectroscopic imaging, positron emission tomography–to–CT, and CT‐to‐CT registrations with an achieved accuracy of 3 mm.^(26)^

One of the main advantages of using the B‐Spline model is that the deformation is interpolated between grid points, making it stable to pixel‐level noise. Unlike other spline models, B‐Spline splines are locally controlled. As a result, displacement of an interpolation point is influenced only by displacement of the closest grid points, and changing a lattice node affects the transformation only regionally, making the model efficient in describing local deformations. The control points are distributed automatically in a uniform mesh overlaid on the image. The user may select the coarseness of the grid, but may not directly place control points in specific locations, such as on structure boundaries.

We selected the Mattes^(22)^ implementation of the mutual information metric because of its speed, simplicity, and stability to noise in the input images. The Mattes implementation does not use all voxels in the input images, but rather evaluates a random sample of the voxels and uses interpolation to evaluate the joint histogram, creating histograms that are less noisy if used as criteria for the optimization.^(22)^ Because only a percentage of the input image voxels are used, the metric is faster than metrics that are evaluated on an entire image.

The parameters of the Mattes metric are the number of samples, expressed as percentages of the total voxels in the input images, and the number of bins used to estimate the joint histogram. For the registration tests in our study, we use settings of 50 histogram bins and 10 percentage samples. The L‐BFGS (limited‐memory Broyden–Fletcher–Goldfarb–Shanno) optimizer^(27)^ was used to find optimal node values, with a maximum of 100 iterations and a maximum of 20 corrections used as termination conditions for the optimization algorithm.

The output of the deformable registration algorithm is a deformation field that correlates each point in the PCT and CBCT images. This information is used to map the contours delineated in the PCT dataset to the CBCT dataset. Starting from a mesh that defines the contour of a critical structure in the PCT dataset, the warping of the contours was implemented by applying the deformation field to modify the coordinates of each point. The vertices were unaltered during the process.

### E. Quantification of registration accuracy

The global accuracy of the deformation field was assessed by creating subtraction images. In the subtraction images, pixel intensity is assigned as the absolute difference between the pixel intensities in the images being compared. Ideally, when the fixed and floating images are identical, the subtraction should be entirely black. Regions of different pixel intensity show disagreement in the input images, identified as regions where the registration has failed. For same‐modality images expected to be identical, the subtraction method provides better accuracy than the commonly used checkerboard, because it visualizes the error difference simultaneously over the entire image.

For the present report, a detailed analysis was performed directly on the contours of the phantom. Because we selected cases of unchanging geometry, structures delineated in the CT image and rigidly translated to the CBCT image will match, because the real‐world deformations are negligible. The non‐deformed structures on either the CT or the CBCT image represent the baseline against which the registration accuracy is to be judged. A copy of the baseline structures is first warped with the random deformation and then warped back by applying the deformation field obtained by the registration. In the ideal case, the deformation field produced by the registration is the exact inverse of the randomly assigned deformation, and thus the successive warpings should produce a null deformation. The result of the deformed and baseline structures should be identical. In practice, noise, artifacts, and registration convergence criteria affect the accuracy of the deformation field deduced by the registration.

Color‐coded surfaces compare the warped and baseline contours and directly quantify the errors produced by the registration. For each node in the baseline contour, a scalar value corresponding to the distance to the nearest point in the registration‐deduced contour is assigned. A color‐coded scale is used to visualize the difference, with blue at one end representing a perfect match and red at the other representing the maximum observed error. The maximum and the mean error are recorded and presented as a quantification of accuracy. Subtraction images of registration results are presented here for qualitative visual inspection of registration accuracy, but we chose to focus our quantitative analysis on the registration of surfaces of contoured structures rather than on the global deformation. We had several reasons for this approach. First, because the phantom itself is largely homogenous, registration errors within regions lacking variability in pixel intensity would go unnoticed. Second, we limited our evaluation to contoured surfaces—an approach that, in our opinion, more closely mirrors a clinically relevant situation.

The chosen approach has these advantages:

- The baseline is known to correspond to the native structures.
- The quantitative value of the registration accuracy takes the form of the mean and maximum errors.
- Direct visualization remains possible in contoured regions where registration fails.

### F. Case studies

The following image data was used for this deformable image registration study:

- A PCT image set of the phantom with 2.5‐mm slice spacing and a pixel size of 0.789×0.789 mm (called *PCT*)
- A PCT set that was artificially deformed ±20 mm (called ${\text{PCT}}_{\text{def}20}$)
- A PCT set which was artificially deformed ±50 mm (called ${\text{PCT}}_{\text{def}50}$)
- A CBCT image set acquired from the OBI system with 2.5‐mm slice spacing and 0.938×0.938 mm pixel size (called *CBCT*)

To evaluate the algorithm's ability to appropriately shift image data, we attempted a registration with the optimization starting from initial known transformation parameters. The following specific data analyses were performed:

- Test 1: The B‐Spline deformable model was used to register the *PCT* image set to the ${\text{PCT}}_{\text{def}20}$ and ${\text{PCT}}_{\text{def}50}$. Because the images were similar except for the artificial deformations, image noise and positioning errors were eliminated. This test was designed to evaluate the selection of convergence parameters for the optimization algorithm, to determine the intrinsic error of the registration algorithm itself, and to compare the ability of the registration algorithm to deal with smaller errors (±20 mm) and larger, less clinically relevant, errors (±50 mm).
- Test 2: The ${\text{PCT}}_{\text{def}20}$ image data was registered to the *CBCT* image set. This test simulated the implementation of ART at our clinic with the planning images being deformed to match the OBI‐acquired CBCT at the time of treatment. The aims of this test were identical to those of the first test, except that the algorithm was being tested by the introduction of image noise in the CBCT image set.

**Figure 2:**
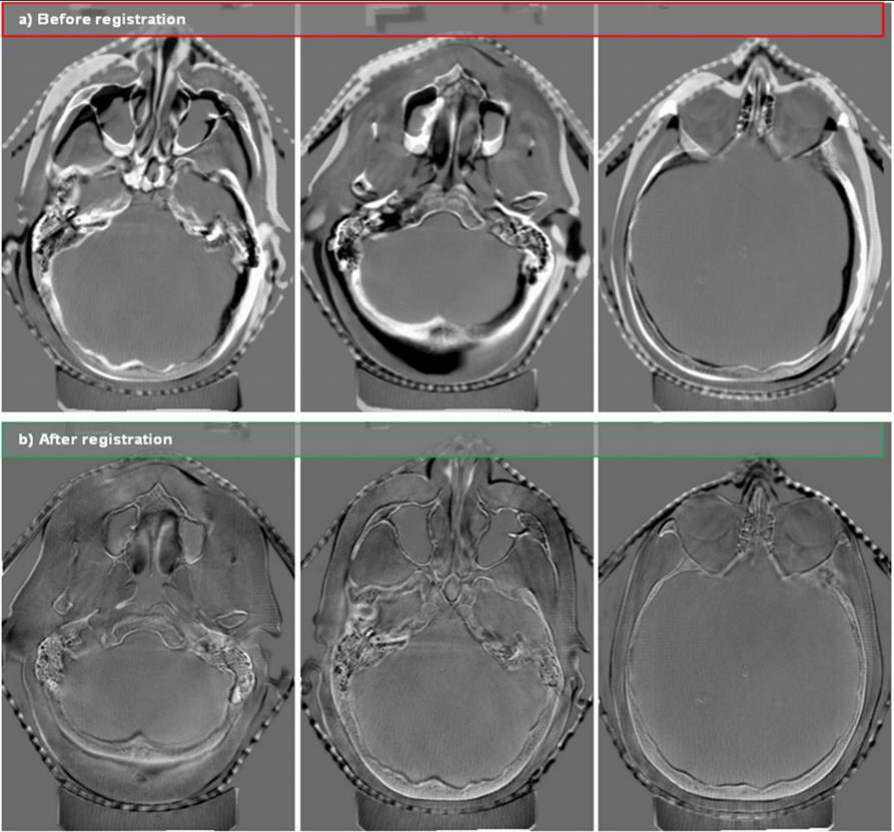
Subtraction images of representative case‐study cone beam computed tomography and planning computed tomography slices (a) before and (b) after registration.

Another analysis using a case study of the brain of a central nervous system (CNS) patient was designed to test the performance of the deformable image registration algorithm. The patient was a 60‐year‐old female with non‐small‐cell lung cancer metastatic to brain. She had been treated previously with stereotactic radiosurgery (SRS), with further SRS to a separate lesion planned. A rigid registration was performed to align the patient's PCT and CBCT image sets for the brain; Fig. 2 shows the result as a subtraction image. This registration provided the baseline accuracy for the system.

After registration, the manually contoured structures on the PCT images were transposed onto the CBCT images and served as a baseline. The contoured structures were the body, brainstem, chiasm, left eye, right eye, medulla oblongata, left optic nerve, right optic nerve, right vermis, and the planning target volume (PTV). As in tests 1 and 2 described earlier, random deformations were applied to the structures to test the ability of the deformable image registration algorithm to return the images to baseline. The random deformations were ±20 mm, because deformations of this magnitude are more reflective of those expected in clinical practice (as opposed to the larger deformations of ±50 mm). Thus, this test was designed to analyze the deformable image registration of the patient's ${\text{PCT}}_{\text{def}20}$ to the original CBCT images acquired with the OBI system.

## III. RESULTS

### A. Study 1(A): PCT‐to‐PCT registration with small deformations

**Figure 3:**
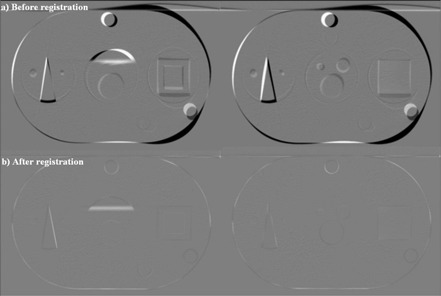
Subtraction images of a computed tomography slice (a) before and (b) after registration to the artificially deformed computed tomography slice with deformations of ±20 mm.

**Figure 4:**
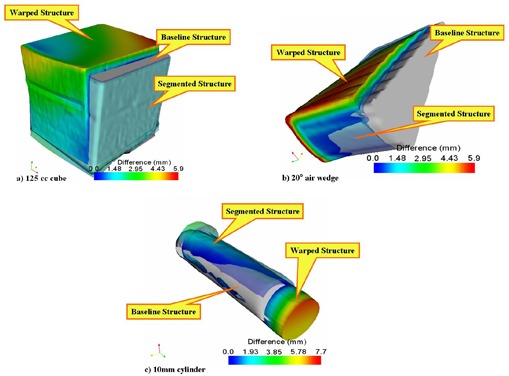
Three images (baseline, warped with ±20‐mm deformations, and registered) of three distinct structures: (a) cube, (b) 20‐degree air wedge, and (c) 10‐mm cylinder. In each image, the gray color denotes the native contoured surface. Overlaid on the native surface are the warped and registered surfaces. The color scale reflects the magnitude of deformation, with red being the maximum deformation. Each image shows the magnitude of introduced deformation and the residual error after registration (light blue color).

Fig. 3(A) shows “before” images of the native PCT and the artificially deformed (±20 mm) PCT. Fig. 3(B) shows subtraction images of the same slices after the registration algorithm was performed. Visual inspection of the subtraction images shows accurate registration, with small visible errors remaining. Table 1 shows the deformation data for each of the 11 contoured structures. After deformation and before registration, the mean of the mean offset for all 11 structures was 2.58 mm (range: 1.70 mm – 3.73 mm), which decreased to 0.34 mm (0.03 mm – 0.74 mm) after registration. The mean of the maximum offset for these deformed structures was 7.39 mm (range: 4.36 mm – 10.24 mm), decreasing to 1.59 mm (0.53 mm – 3.54 mm) after registration. Fig. 4 shows representative images of several structures at baseline, warped, and registered using the algorithm.

Also analyzed were the volumes of each contoured structure in the native PCT image set, after deformation, and after registration. Table 2 presents those results. The mean percentage error introduced by the deformations was 26.6% (range: 1.9% – 52.6%). The mean percentage residual error after registration was 5.5% (range: 0.4% – 14.5%).

**Table 2:** Original, deformed (±20 mm), and registered volumes of each structure for computed tomography (CT)–to–computed tomography registration with small deformations

		Volume (mm^3^)			Percentage	
Structure	Size	On native CT image set	After deformation	After registration	Deformation	Residual error
Body		5528440.00	5424890.00	5507300.00	1.9	0.4
Cube	125 cm^3^	110730.00	77033.90	118048.00	30.4	6.6
Sphere	40 mm	34939.90	32801.70	37835.80	6.1	8.3
	20 mm	3652.83	2833.96	3969.21	22.4	8.7
	10 mm	519.24	282.09	530.93	45.7	2.3
Air wedge	20 degrees	49701.70	36241.30	50278.80	27.1	1.2
	60 degrees	199465.00	124651.00	197048.00	37.5	1.2
Cylinder	10 mm	3759.82	5735.69	4305.43	52.6	14.5
	5 mm	906.91	718.04	871.36	20.8	3.9
Solid rod	25 mm	28326.10	18978.70	30008.40	33.0	5.9
Air rod	25 mm	32128.90	27134.00	29744.30	15.5	7.4

### B. Study 1(B): PCT‐to‐PCT registration with large deformations

**Figure 5:**
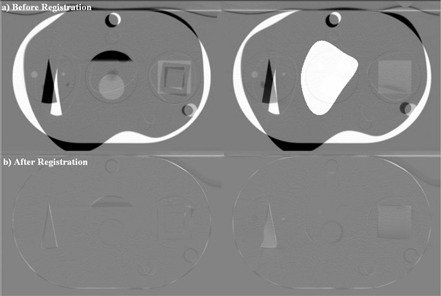
Subtraction images of a computed tomography slice (a) before and (b) after registration to the artificially deformed computed tomography slice with deformations ±50 mm.

**Figure 6:**
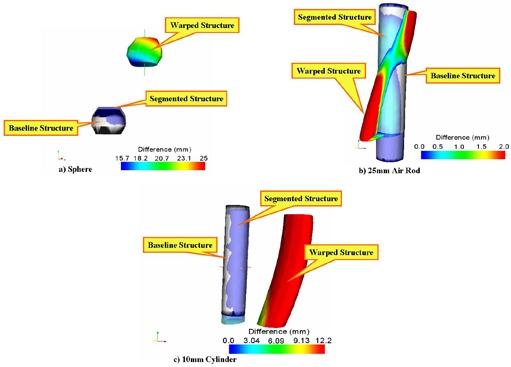
Three representative surfaces (baseline, warped, and segmented) of (a) a sphere, (b) a 25‐mm air rod, and (c) a 10‐mm cylinder. In each image, the native surface is shown in gray. The color scale reflects the magnitude of introduced deformations, with red being the maximum deformation. The third, registered, surface shows the residual error following image registration.

(Fig. 5A,B) shows “before” and “after” subtraction images of the native PCT image and the deformed PCT image with larger deformations of ±50 mm. Visual inspection again reveals an accurate registration of the native and deformed images, with only small remaining visible discrepancies. Table 3 shows the mean and maximum directional offset for each of the 11 structures after deformation and after subsequent registration. The mean of the mean offset after deformation was 10.51 mm (range: 2.57 mm – 20.61 mm), which decreased to 0.47 mm (0.11 mm – 0.95 mm) after registration. The mean of the maximum offset introduced by the deformation was 19.90 mm (range: 7.86 mm – 28.30 mm). Implementation of the registration algorithm reduced this to offset to 3.94 mm (0.83 mm – 10.50 mm). Fig. 6 shows representative images of several structures at baseline, deformed, and registered.

**Table 3:** Mean and maximum structure deformations introduced and remaining after registration for artificial deformations of ±50 mm

		Offset (mm)			
		After deformation		After registration	
Structure	Size	Mean	Maximum	Mean	Maximum
Body		2.60	18.26	0.11	10.18
Cube	125 cm^3^	3.83	11.45	0.95	10.50
Sphere	40 mm	13.77	28.30	0.64	1.81
	20 mm	16.49	24.41	0.35	1.00
	10 mm	20.61	25.57	0.45	0.83
Air wedge	20 degrees	9.72	24.30	0.49	2.06
	60 degrees	9.55	20.36	0.34	2.29
Cylinder	10 mm	18.15	27.73	0.68	4.19
	5 mm	15.46	18.46	0.51	1.89
Solid rod	25 mm	2.90	12.17	0.22	1.86
Air rod	25 mm	2.57	7.86	0.43	6.72

Volume analysis was again performed (Table 4). The mean volume error as a result of the deformation was 26.64% (range: 1.87% – 52.55%), which decreased after registration to a mean percentage residual error of 5.49% (0.38% – 14.51%). The residual errors for the ±20 mm deformations and the ±50 mm deformations are similar, suggesting that registration accuracy does not depend on the magnitude of the deformation.

**Table 4:** Original, deformed (±50mm), and registered volumes of each structure for computed tomography (CT)–to–computed tomography registration with large deformations

		Volume (mm^3^)			Percentage	
Structure	Size	On native CT image set	After deformation	After registration	Deformation	Residual error
Body		5528440.00	5424890	5507300	1.87	0.38
Cube	125 cm^3^	110730.00	77033.9	118048	30.43	−6.61
Sphere	40 mm	34939.90	32801.7	37835.8	6.12	−8.29
	20 mm	3652.83	2833.96	3969.21	22.42	−8.66
	10 mm	519.24	282.093	530.927	45.67	−2.25
Air wedge	20 degrees	49701.70	36241.3	50278.8	27.08	−1.16
	60 degrees	199465.00	124651	197048	37.51	1.21
Cylinder	10 mm	3759.82	5735.69	4305.43	−52.55	−14.51
	5 mm	906.91	718.037	871.361	20.83	3.92
Solid rod	25 mm	28326.10	18978.7	30008.4	33.00	−5.94
Air rod	25 mm	32128.90	27134	29744.3	15.55	7.42

### C. Study 2(A): PCT‐to‐CBCT registration

**Figure 7:**
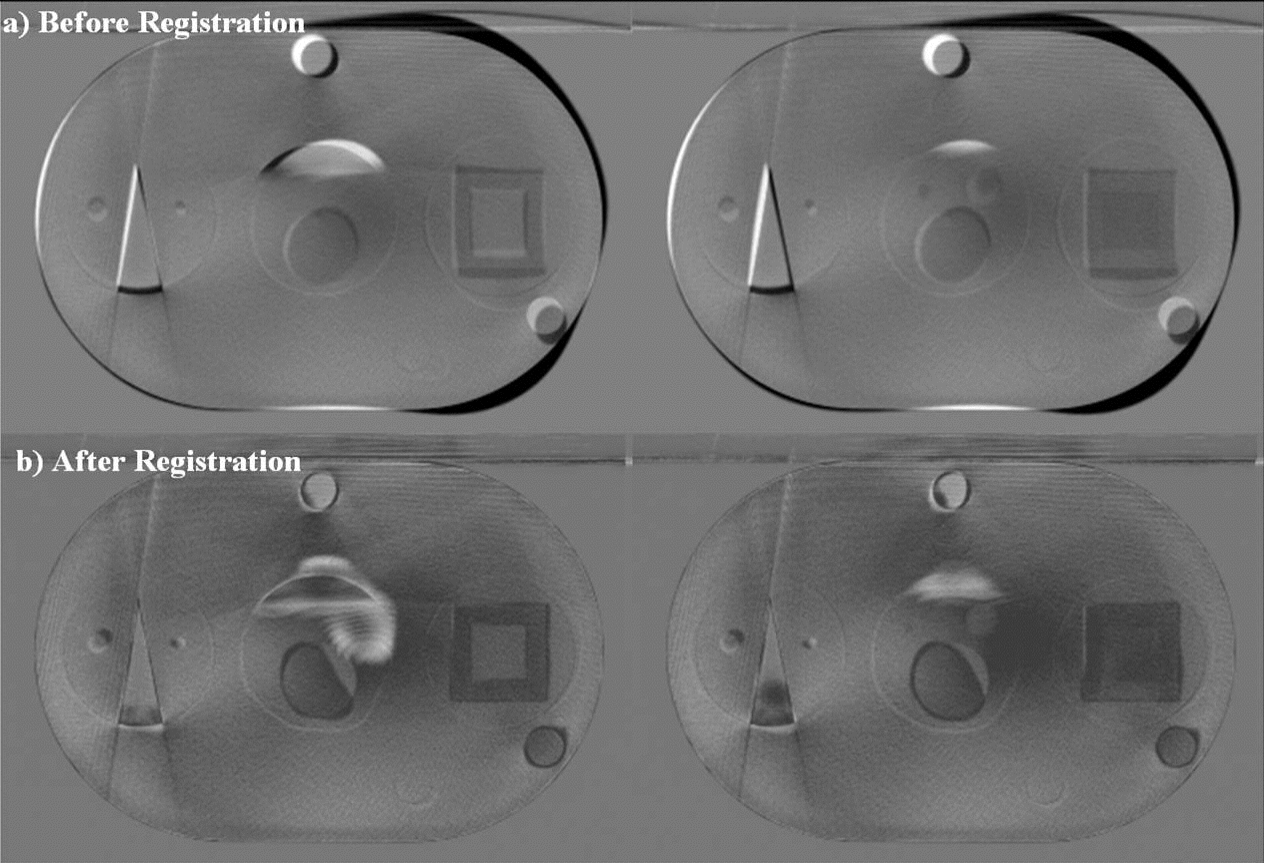
Subtraction images of a cone‐beam computed tomography (CBCT) slice (a) before and (b) after registration to the artificially deformed computed tomography slice with deformations of ±20 mm. These images show the noise present in the CBCT image set.

The second component of the analysis was designed to test the performance of the registration algorithm in the presence of image noise. Fig. 7 shows subtraction images before and after deformations of ±20 mm were introduced as outlined earlier. Subtraction images are not preferred for intermodality registrations, and visual inspection shows substantially more noise in the images, but still a visually accurate registration is seen. As before, Table 5 shows the mean and maximum offset for each of the structures after deformation and after registration. The mean of the mean offset introduced by the deformation was 2.93 mm (range: 1.45 mm – 4.51 mm); the mean offset decreased to 1.33 mm (0.69 mm – 2.47 mm) after registration. After deformation, the mean of the maximum offset was 8.76 mm (range: 6.70 mm – 11.21 mm), which decreased to 4.86 mm (1.75 mm – 7.75 mm) after registration. Residual maximum deformations were larger than those seen with smaller introduced deformations.

**Table 5:** Mean and maximum structure deformations introduced and remaining after computed tomography (CT)–to–cone‐beam computed tomography registration for artificial deformations of ±20 mm

		Offset (mm)			
		After deformation		After registration	
Structure	Size	Mean	Maximum	Mean	Maximum
Body		1.45	10.18	1.08	5.73
Cube	125 cm^3^	3.50	9.88	1.02	4.73
Sphere	40 mm	4.18	9.31	1.61	7.29
	20 mm	2.70	6.70	1.71	5.71
	10 mm	3.63	6.76	2.47	7.75
Air wedge	20 degrees	3.88	9.35	1.56	7.47
	60 degrees	2.00	9.36	0.69	1.75
Cylinder	10 mm	1.75	7.07	1.16	4.36
	5 mm	4.51	11.21	1.00	2.50
Solid rod	25 mm	2.09	8.08	1.14	2.92
Air rod	25 mm	2.57	8.44	1.15	3.25

Table 6 presents the volume analysis. The mean volume error introduced by the deformations was 7.73% (range: 0.47% – 18.73%); the mean residual volume error after registration was 18.63% (0.01% – 95.63%). The largest residual errors were seen for the smallest structures, with the worst outcome for the 10‐mm sphere.

**Table 6:** Original, deformed (±20 mm), and registered volumes of each structure for cone‐beam computed tomography–to–computed tomography registration

		Volume (mm^3^)			Percentage	
Structure	Size	On Native CT image set	After deformation	After registration	Deformation	Residual error
Body		5528440.00	5502270.00	5448970.00	0.5	1.4
Cube	125 cm^3^	110730.00	91861.30	108950.00	17.0	1.6
Sphere	40 mm	34939.90	34744.00	30708.70	0.6	12.1
	20 mm	3652.83	3841.25	1898.75	5.2	48.0
	10 mm	519.24	543.63	22.69	4.7	95.6
Air wedge	20 degrees	49701.70	54237.30	42334.10	9.1	14.8
	60 degrees	199465.00	170745.00	199482.00	14.4	0.0
Cylinder	10 mm	3759.82	3904.34	3360.67	3.8	10.6
	5 mm	906.91	911.81	792.58	0.5	12.6
Solid rod	25 mm	28326.10	23019.90	29783.00	18.7	5.1
Air rod	25 mm	32128.90	35486.60	31188.60	10.5	2.9

### D. Study 2(B): Case study of CNS patient

**Figure 8:**
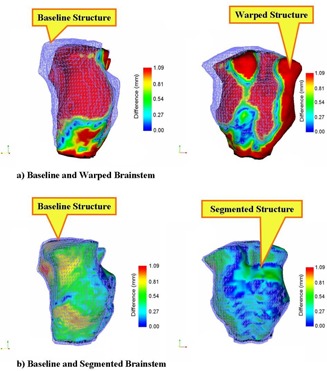
Representative sample of cone‐beam computed tomography (CBCT)–to–planning computed tomography registration in a clinical case, showing (a) the native and (b) the deformed contoured brainstem surfaces. The color scale shows the magnitude of the deformation, with blue and red respectively representing areas of least and farthest distance between the native and deformed surfaces. A comparison of panels A and B highlights the ability of the algorithm to accurately recover the native surface.

Because the CBCT‐to‐PCT registration showed increased error in imaging a phantom, we used a patient test case to evaluate the algorithm (Table 7). In contrast to CBCT‐to‐CT registration using phantom image sets, the linear offsets remaining after registration of the case study images were quite small. For the contoured structures, the mean of the mean residual offset was 0.69 mm (range: 0.34 mm – 1.70 mm). The mean of the maximum residual offset for these structures was 2.00 mm (range: 0.72 mm – 8.13 mm). The maximum residual offset was >1.5 mm in only three structures: the body (8.13 mm), the left eye (1.71 mm), and the right front prior field (3.48 mm). The mean residual volume discrepancy was 4.97% (range: 0.82% – 11.62%). Fig. 8 shows representative native, deformed, and registered structures.

**Table 7:** Following deformation and registration to original computed tomography images, residual linear errors and residual volume errors for contoured structures

	Error after registration (mm)		Volume (mm^3^)		
Structure	Mean	Maximum	After registration	As originally contoured	Percentage residual volume error
Body	1.05	8.13	3.48E+06	3.40E+06	2.3
Brainstem	0.39	1.09	21959.1	23189.6	5.6
Chiasm	0.47	0.72	152.504	155.767	2.1
Eye, left	0.64	1.71	10269.9	9646.22	6.1
Eye, right	0.40	1.14	10886.8	10732.8	1.4
Medulla oblongata	0.55	1.08	3274.48	3247.99	0.8
Optic nerve left	0.68	1.37	234.783	240.979	2.6
Optic nerve right	0.34	0.72	260.88	270.36	3.6
Rt. front prior field	1.70	3.48	255.177	228.619	10.4
Rt. vermis	0.67	1.30	128.881	141.983	10.2
PTV	0.66	1.29	330.167	363.729	10.2

PTV=planning target volume.

## IV. DISCUSSION

Radiation therapy has progressed in recent years, evolving from treatment with large fields encompassing both tumor and at‐risk neighboring areas to increasingly conformal treatment for many disease sites. As this progression from conventional radiotherapy to 3D‐conformal radiotherapy to intensity‐modulated radiation therapy to IGRT continues, emphasis is increasingly being placed on the precision of imaging and registration. To successfully incorporate IGRT, an accurate and rapid image registration technique is required. Although rigid registration can facilitate accurate registration of a small volume, the technique is typically unable to accurately register entire image sets because of variations in patient positioning and imaging technique, and alterations in internal soft‐tissue structures including tumor and normal tissues alike. As efforts to develop ART escalate, the ability to alter manually segmented volumes to reflect daily changes in shape and location has become increasingly important.

We used an automated quantitative evaluation method to test a deformable image registration algorithm in several phantom cases and a clinical case. Initial testing of the registration of original CT images to artificially warped CT images showed a maximum intrinsic algorithm error of approximately 2 mm, which we judge acceptable. Also, the optimization did not depend on the magnitude of the deformation: the residual errors after registration were similar, despite a large difference in the magnitudes of the deformation introduced. The mean residual error was also acceptable, with a mean residual error of <1 mm (thought to be the result of tolerance in the algorithm). Attempted PCT‐to‐CBCT registration introduced image noise and produced results that were less accurate, indicating algorithm sensitivity to noise (most pronounced for small structures). Residual error in surface deformation and in structure volume was larger than that seen the previous test, with remaining maximum offsets of more than 2 mm in all but one contoured structure. Still, the mean residual error after registration was less than 2 mm for all but one structure. These larger residual errors were thought to be attributable to a combination of air adjacent to the first and last CBCT slices and of the HU homogeneity of the phantom structures. Both of these potential sources of error are somewhat mitigated in the case of a patient as opposed to a phantom. The structures within a patient are far less homogenous in HU value, and the first and last image slices are adjacent to tissue instead of air. The CBCT images in Fig. 7 highlight the image noise adjacent to the structures within the phantom image set. Markedly fewer artifacts are seen in patient CBCT image sets.

Considering the foregoing factors, we next conducted a case study using images of a patient treated for a CNS cancer to assess the robustness of the registration algorithm in a clinically realistic setting. We found that, as with the initial CT‐to‐CT phantom image registration, the registration algorithm was stable to the amount of noise present in the patient's CBCT and produced excellent results, with residual maximum offsets of approximately 2 mm. Fig. 8 shows the results, with excellent correlation of the native and segmented surfaces. The algorithm's performance was worse for the right frontal prior field structure. That finding was expected, because that particular structure was not anatomically defined, but instead arbitrarily transferred from a previous treatment plan, so that its borders are in areas of HU homogeneity, which limits the performance of the algorithm.

Typical expansions from the clinical target volume (CTV) to the PTV are 1 – 1.5 cm for most disease sites and patient immobilization techniques. This expansion allows for intrafraction and interfraction target movement and daily setup error. We are able to acquire and process a CBCT image set in approximately 60 seconds. In each case, the registration process adds another 5 – 7 minutes (varying with the number of iterations). With an intrinsic error of approximately 2 mm and the ability to rapidly acquire, process, and register images and structures, the implications of the technique described here are encouraging.

Interest in ART has grown out of the desire to reduce required margins for error, to target movement, and potentially to improve the therapeutic ratio by increasing the amounts of normal structures spared. Truly adaptive therapy will need to change from fraction to fraction to reflect daily changes in patient anatomy. Using the technique reported here, it would be possible to quickly and accurately acquire and register daily CBCT image sets with the CT image set generated at the time of simulation. It is our hope that such registration will lead to the ability to make daily online alterations in treatment delivery to reflect known anatomic changes just before delivery of that day's fraction.

We chose to estimate the registration on the contour surfaces directly rather than use the standard difference and checkerboard tools because we believe that the surface‐based evaluation gives better accuracy. Different solutions to any deformable problem may exist, because the deformation field inside a structure of uniform intensity can be of any value. Only the voxels that lie on structure borders contribute to the cost function and its derivative. Voxels mapped by the deformation algorithm within the same homogenous structure do not contribute to the cost function and thus are indiscernible clinically or by the optimization algorithm. The deformation inside a homogenous structure can therefore have any value, as long as it does not cross that structure's border, because voxels from inside the structure will map to the structure itself. For example, in the image background, the deformation field can have (and does have) any value, because air will deform to air.

The optimization algorithm does not detect changes in the cost function when modifying the values in the B‐Spline located in free air, thus retaining the initial values. Non‐zero deformations in regions of varying intensities would be an error, because those deformations would map voxels from one structure to another. Thus, a global assessment of the deformation field is possible, as previously, but will provide only a quantitative evaluation of the deformation field. The accuracy lies in the details at structure borders, because solution values are irrelevant elsewhere. As opposed to using subtraction images to asses the “global” deformation field, the new surface‐based method permits calculation of deformation field errors with subvoxel accuracy, simplifies the analysis (because it takes into account only the clinically relevant regions), and provides user‐independent measures such as mean and maximum error computed directly on the surface. The deformation map deduced on the grid of control points can then be applied on the dose matrix for ART.

The evaluation method presented here is independent of the deformable registration model used. Several computational registration models have been proposed, with the applicability of each model to a particular registration problem depending on and being restricted to the type of assumptions made concerning the nature of the images to be registered. The fluid‐flow approach,^(^ ^28^ ^–^ ^32^ ^)^ B‐Spline,^22^ ^,^ ^24^ ^,^ ^26^ and the finite element approach^(^ ^33^ ^–^ ^36^ ^)^ are the ones most commonly used. Because the evaluation method presented here directly compares the contours obtained by warping with the deformation field, it can easily be applied to evaluate the accuracy of methods other than the B‐Spline model. Minimal changes to the standard code are needed to implement random artificial deformation. The resulting experiments minimize human error during acquisition and bias in the evaluation of results. Although the present works focuses on CBCT‐to‐CT registration for ART, the evaluation method is general and can be applied to any type of deformable registration. By selecting different input configurations to the registration algorithm, detailed information can be independently obtained on the errors induced by the various factors influencing accuracy.

## V. CONCLUSION

We showed that a deformable image registration technique was accurate and stable to the amount of noise present in CBCT images of a patient. However, to realize the full potential of adapting radiotherapy fractions just before delivery (online approach), registration will need to occur quickly to facilitate daily changes in fraction delivery made to reflect daily changes in patient anatomy. Efforts at our institution and others are currently underway.
